# Timing Selector: Using Transient Switching Dynamics to Solve the Sneak Path Issue of Crossbar Arrays

**DOI:** 10.1002/smsc.202100072

**Published:** 2021-10-10

**Authors:** Mingyi Rao, Wenhao Song, Fatemeh Kiani, Shiva Asapu, Ye Zhuo, Rivu Midya, Navnidhi Upadhyay, Qing Wu, Mark Barnell, Peng Lin, Can Li, Zhongrui Wang, Qiangfei Xia, J. Joshua Yang

**Affiliations:** ^1^ Department of Electrical and Computer Engineering University of Massachusetts Amherst Amherst MA 01003 USA; ^2^ Ming Hsieh Department of Electrical and Computer Engineering University of Southern California Los Angeles CA 90089 USA; ^3^ Air Force Research Lab Information Directorate Rome NY 13441 USA

**Keywords:** crossbar arrays, memristors, neuromorphic computing, resistive switching, selectors

## Abstract

Sneak path current is a fundamental issue and a major roadblock to the wide application of memristor crossbar arrays. Traditional selectors such as transistors compromise the 2D scalability and 3D stack‐ability of the array, while emerging selectors with highly nonlinear current–voltage relations contradict the requirement of a linear current–voltage relation for efficient multiplication by directly using Ohm's law. Herein, the concept of a timing selector is proposed and demonstrated, which addresses the sneak path issue with a voltage‐dependent delay time of its transient switching behavior, while preserving a linear current–voltage relationship for computation. Crossbar arrays with silver‐based diffusive memristors as the timing selectors are built and the operation principle and operational windows are experimentally demonstrated. The timing selector enables large memristor crossbar arrays that can be used to solve large‐dimension real‐world problems in machine intelligence and neuromorphic computing.

## Graphical abstract

A timing selector is proposed, which can utilize its nonlinear relation between switching time and voltage (instead of current and voltage in traditional selectors) to block sneaking current in memristive crossbar arrays. The operation principles are formulated together with experimental demos using integrated one‐selector‐one‐memristor (1S1M) structures.

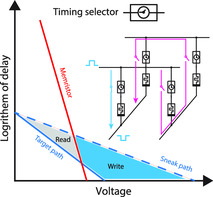

## 1 Introduction

A biological neural network may be emulated by solid‐state devices connected to a crossbar array,^[^ 1, 2, 3, 4, 5 ^]^ in which conductances of such devices represent the synaptic weights and the weighted sum of electric current implements the multiply and accumulate (MAC) operations. The synaptic devices should have voltage‐induced programmability in conductance. Voltage‐induced resistive switching phenomenon has been observed in a variety of materials, giving rise to different types of neuromorphic devices.^[^ 6, 7, 8, 9, 10, 11, 12, 13 ^]^ Among these devices, memristors, especially the bipolar oxide‐based memristors, are highly promising due to their excellent scalability, endurance, retention, and multilevel resistance.^[^ 14, 15, 16, 17, 18 ^]^ A major challenge to implement an artificial neural network with large‐scale crossbar arrays of two‐terminal memristive devices is the so‐called “sneak path” problem, meaning unwanted current flows through other devices when a voltage is applied across a target device.^[^ 19, 20, 21, 22 ^]^ The existence of sneak path currents makes reading and writing a cross‐point device (target device) inaccurate or inefficient. To address the problem, either the memristor needs to be engineered, or a select device should be added in series with the memristor, to obtain a highly nonlinear current–voltage relation in a cell. As a result, the sneak path currents are negligible compared with the current flowing through the target cell. The former can be realized by designing a material stack of memristors so that its current–voltage relation is highly nonlinear or self‐rectified.^[^ ^23^ ^]^ In the latter method, a diode,^[^ ^24^ ^]^ a transistor,^[^ ^14^ ^]^ or a threshold switching device^[^ 25, 26 ^]^ can serve as a select device.

In contrast, to accurately compute the weighted sum of presynaptic inputs, a linear current–voltage relation is required for each cell, contradicting the nonlinearity requirement to suppress the sneak path current. Transistors have been the most successful select devices to date as the current can be precisely tuned through the gate during cell writing, whereas a linear current–voltage relationship is maintained by operating the transistor in the triode region during readout.^[^ ^27^ ^]^ However, transistors are reaching the scaling limit, and their fabrication process and three‐terminal configuration impose challenges for 3D stacking, a pathway forward for future complex neural networks with massive connectivity.^[^ ^28^ ^]^ Two‐terminal select devices based on nonlinear current–voltage behavior, although having been extensively studied and developed, only have limited success so far for the application in large‐scale arrays.

In this article, we report a type of select device, coined timing selector, that takes advantage of the nonlinear relation between delay time and voltage of the device rather than the nonlinear relation between the current and voltage. The delay time of the selector refers to the incubation time needed to turn on such a device when a voltage pulse is applied and is reversely related to the amplitude of the voltage pulse across the device. By applying a larger voltage to the target device/timing selector pair, the pertinent selector is turned on, whereas those in the sneak paths are not, enabling programming or reading the target cell without disturbing the neighbors. We experimentally demonstrated the concept with silver‐based diffusive memristors. We further identified the operational window of memory cell with such selectors and extended the timing selector concept to other resistive switching devices with similar transient dynamics. The timing selector performance was verified in crossbar arrays from 2 × 2 to 64 × 64 experimentally and the device is expected to work in a crossbar array with ≈10^7^ columns (rows) according to analysis. The timing selector is fully compatible with artificial neural networks and will pave the way for the wide application of large‐scale memristive crossbar arrays for nontraditional computing applications.

## 2 Results

**Figure 1:**
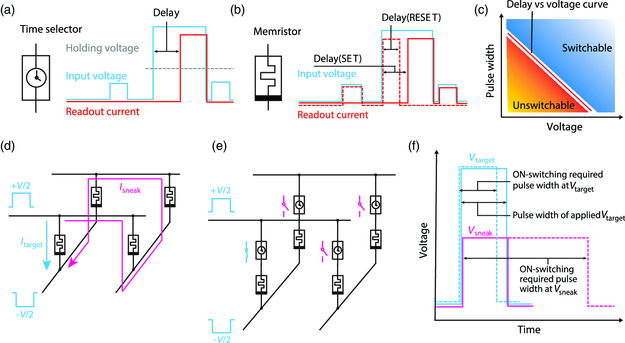
The sneak path blocking mechanism of a timing selector. a) The symbol and required electrical characteristics of a timing selector. The blue and red curves represent the absolute value of input voltage and readout current. A timing selector can be switched to LRS from its initial HRS after being exposed to voltage with sufficient amplitude for the “delay” time. Once the voltage amplitude drops below a certain value (the “holding voltage”), the device will switch to its HRS spontaneously. b) The symbol and typical characteristics of a memristor. The blue and red curves represent the absolute value (whereas opposite polarities are normally used for SET and RESET) of input voltage and readout current. The resistive switching (SET or RESET) also takes a “delay” time to occur. A memristor can maintain its resistance state after the external voltage is removed. c) The response to a voltage pulse of a timing selector. It is required that the delay of a timing selector decreases with increasing voltage amplitude. Their relationship is represented by the delay–versus–voltage curve, which is also referred to as the delay curve. If an applied voltage pulse's amplitude and width correspond to a point above the delay curve of the timing selector, the timing selector can be switched to LRS from its initial HRS. d) A schematic of sneak path current in a crossbar array formed by memristors. When voltage pulses of amplitude *V*/2 with opposite polarities are applied to two electrodes of the target memristor, there is current flowing through both the target device (*I*_target_) and other devices (*I*_sneak_) in the array that forms a conductive path in parallel with the target device. e) A schematic of the timing selector serves as a voltage‐controlled switch that blocks the sneak path current while providing access to the target device. f) The sneak path blocking mechanism in (e). Because each timing selector in the sneak path shares a relatively smaller voltage *V*_sneak_ compared with the target timing selector voltage *V*_target_, the delay of a sneak path selector is longer than that of the target selector. If the voltage pulse is chosen so that the pulse width is longer than the timing selector's delay at *V*_target_ but shorter than the timing selector's delay at *V*_sneak_, the target selector will be switched to its LRS while sneak path selector will maintain its off state.

The symbols and required electrical behaviors of a timing selector and a memristor are shown in **Figure** **1**a,b, respectively. A major difference between them is that the timing selector is volatile and the memristor is non‐volatile. In other words, the memristor can maintain its high‐/low‐resistance state (HRS/LRS) when zero voltage is applied, whereas a timing selector always remains in its HRS unless it receives an external stimulus (e.g., a voltage pulse), which is sufficient to switch it to its LRS. If the external voltage drops below a certain holding voltage, the selector will spontaneously change back to its HRS. The volatile‐resistive switching behavior negates the need for extra operations to mute a one‐selector‐one‐memristor (1S1M) unit when it is not selected anymore, which reduces the energy consumption and the risk of inadvertently changing the information stored in the memristor during such operations. The electrical behavior of a timing selector should also be nonpolar so that both positive and negative voltages can switch it on to ensure its compatibility with bipolar memristors.

Besides being volatile and nonpolar, the resistance levels of a timing selector must follow certain criteria. First, a timing selector should work in a binary manner. If a device features apparent analog behavior (its resistance can be gradually tuned in many levels in a wide range), it is not suitable to be a selector because it increases the complexity of reading and writing both selectors and their connected memristors. Second, the off‐state resistance of a timing selector is required to be much higher than that of a memristor, so that the input voltage would mainly drop on the selector before the selector is switched on. In fact, it should be as high as possible so that the total sneak current through all off‐state selectors is negligible compared with the target path current through the target device(s). Third, the on‐state resistance of the selector should be as low as possible that once the selector is switched on, its resistance is ideally negligible to ensure a sufficient voltage drop on the memristor to read or program it.

A schematic illustrating the response of a timing selector under a voltage pulse with variable amplitude and width is shown in Figure 1c. It is required that the time delay upon a voltage to switch the selector is shorter when the voltage amplitude is larger so that the target selector (subject to a higher voltage amplitude) can be switched on much earlier than the selectors on the sneak paths. It can be inferred that only when the amplitude and width of a voltage pulse correspond to a point above the delay curve (red line in Figure 1c), the selector is switched on. An example of sneak path current in a two‐by‐two crossbar with memristors is shown in Figure 1 d, assuming that the unselected bitline (BL) and wordline (WL) are floating. If the unselected BL and WL are grounded, only devices sharing the same BL or WL with the target device can form sneak paths. Sneak current in a large array can always be viewed as a combination of sneak path currents in many 2 × 2 arrays. Figure 1e shows the states of timing selectors in a memristor crossbar array when the applied pulse meets the requirement shown in Figure 1f. When voltage *V* is applied to the target 1S1M cell, no matter the unselected BL and WL are floating or grounded, the 1S1Ms in the sneak paths are under voltage(s) always smaller than that of the target 1S1M. As schematically shown in Figure 1c, the time delay of the target selector is expected to be smaller than those of the sneak path selectors. Because the pulse width is the same for both the target device and sneak path devices, if the pulse width of applied voltage *V*_target_ is chosen to be longer than the delay of *V*_target_ (defined by the red line of Figure 1c), but shorter than the delays of the sneak device voltages (*V*_sneak_), the selectors in the sneak path will not be switched on while the target selector will be.

**Figure 2:**
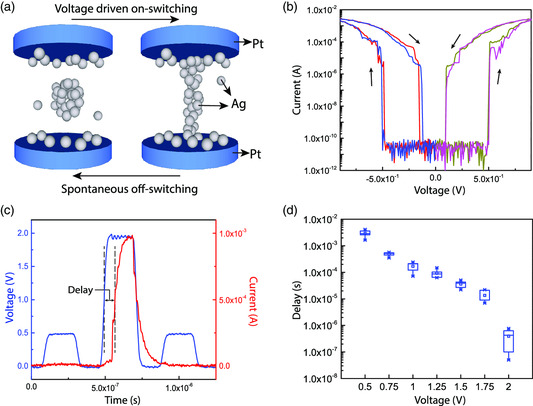
The device structure, mechanism, and electrical characteristics of a timing selector. a) Schematics of device structure and switching mechanism of a timing selector. The device has a Ag‐doped SiO_2_ layer sandwiched between two Pt electrodes. The switching from a HRS to LRS (on‐switching) is the result of Ag particles forming a conductive filament between two electrodes under an electric field. The switching from LRS to HRS (off‐switching) occurs when voltage is removed, which is the result of Ag filaments reshaped into ball‐like structures due to surface energy minimization. b) Voltage sweeping measurement result of a timing selector. When the amplitude of voltage reaches more than 0.5 V, the device can be switched from ≈10 GΩ to less than 1 kΩ. When the voltage amplitude drops below 0.1 V, off‐switching takes place. c) Device reading and switching by voltage pulses. A 2 V pulse can switch a timing selector on within a delay time of less than 100 ns. The device then switches off within 200 ns after the falling edge of the 2 V pulse, according to the 0.5 V pulse postreading. d) The statistics of delay time when a timing selector is under different voltages.

A Ag filament‐based resistive switching device was fabricated as a timing selector to demonstrate the principles as this type of device has proven nonpolar volatile resistive switching, extremely a high ON/OFF ratio, voltage‐dependent switching speed, and endurance.^[^ 25, 29 ^]^ The schematics of the device structure and resistive switching mechanism are shown in **Figure** **2**a, where a Ag‐doped SiO_2_ layer is sandwiched between two Pt electrodes. Fabrication details of the device are described in the Experimental Section. The ON switching of the selector was induced by voltage‐driven Ag‐ion movements, resulting in the formation of a conduction filament(s).^[^ 30, 31 ^]^ When the applied voltage on the device decreases below the holding voltage *V*_hold_, the thread‐like conductive filament spontaneously reshapes itself into a sphere to minimize its surface area and thus interfacial energy between the Ag metal and the dielectric material, which ruptures the filament and leads to OFF switching.^[^ ^9^ ^]^

Electrical testing has been conducted to verify if such devices meet all requirements for a timing selector. The voltage‐driven resistive switching behavior of a timing selector was measured by DC sweeps, as shown in Figure 2b. Due to the symmetric structure of the selector, the current–voltage relationship also showed no voltage polarity dependence. The device could be switched from more than 10 GΩ intrinsic resistance to a few hundred ohms by less than 1 V quasi‐DC voltage sweeps and would switch to HRS once the voltage sweep goes back to near zero. The switching speed of a selector was measured by voltage pulse testing experiments, as shown in Figure 2c. A piece of measurement consisted of a prewriting pulse to read the state before writing, a writing pulse with higher amplitude, and a postwriting pulse to read the state after writing. It was observed the current started to increase abruptly after a delay time. The delay was caused by Ag oxidation, ion migration, and nucleation during the formation of a conduction filament.^[^ ^32^ ^]^ The measured delay times decreased exponentially with amplitudes of the applied voltages, as shown in Figure 2d. The reason is that the mobility of ions is exponentially dependent on the energy barrier to hop through, and such a barrier is effectively lowered by the applied electric field.^[^ ^33^ ^]^ The nonpolar switching behavior, high ON/OFF ratio, and negative slope of delay–voltage relationship suggested that such devices possess all essential features of a timing selector. A memristor with a material stack of Pt (bottom electrode)/HfO_2_/Ta (top electrode) was found compatible with the Ag‐based timing selector in terms of operating voltages and resistance. The device material stack and DC sweep of such memristors is shown in Figure S1, Supporting Information. Note that its HRS is ≈10 kΩ, which is more than six orders of magnitude smaller than that of the HRS resistance of the Ag‐based timing selector, meaning that majority of the applied voltage always drops on the selector before it is switched on.

**Figure 3:**
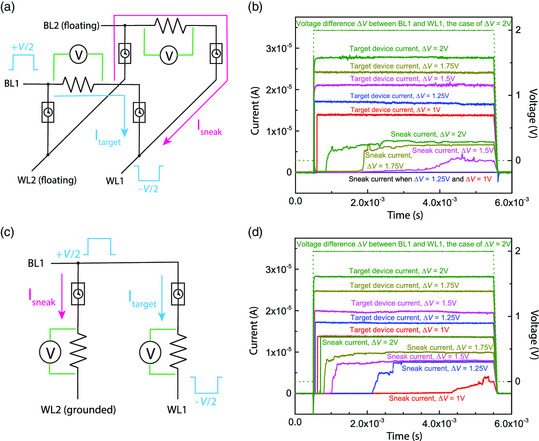
The delay comparison between the target path and the sneak path in timing selector arrays. a) The schematic of the first setup where the untargeted BL and WL are floating. In a 2 × 2 crossbar array formed by connecting multiple individual timing selectors and 68 kΩ standard resistors by cables and breadboard, voltage pulses with an amplitude of *V*/2 with opposite polarities are applied to BL1 and WL1. The target and sneak path current are measured by reading the voltages drop on the resistors. b) Response of the target and sneak paths to a 5 ms voltage pulse in (a). The current is plotted by solid lines, and the input voltage is plotted by a dashed line. The rising/falling edges of all voltage pulses (*V* = from 1 to 2 V) are at the same time, and only the *V* = 2 V pulse is plotted. Sneak path delay decreases with increasing *V*, but it is still longer than the delay of the target path. When *V* = 1 or 1.25 V, the sneak path is not switched on within 5 ms. c) The schematic of the second setup where the untargeted BL and WL are grounded. In this setup, the sneak path current only exists in devices sharing the same BL or WL with the target device, and the voltage on a sneak path device is *V*/2. d) Response of the target and sneak paths to a 5 ms voltage pulse in (c). The current and voltage are plotted in the same way as in (b). It is noticed that the sneak path delay is shorter than the set up in (a), which is due to the higher voltage drops on a sneak path device.

A two‐by‐two crossbar array of timing selectors was built to verify the sneak path current mitigation mechanism, as shown in **Figure** **3**. Memristors were not included in this setup to better illustrate the working mechanisms of the selectors. In the first setup, as shown in Figure 3a, the target selector was the one between BL1 and WL1. The sneak path was formed by the other three selectors. To monitor the current in the target path and sneak path simultaneously, two 68 kΩ standard resistors were introduced to the array. The currents through the two resistors could be calculated by their measured voltages. A positive voltage pulse with the amplitude *V*/2 and a negative voltage pulse with the same amplitude was applied to BL1 and WL1, respectively. The pulse width was 5 ms. BL2 and WL2 were floating. The currents through the target device and the sneak path were measured when various voltages from 1 V to 2 V were applied and are shown in Figure 3b. It was observed that when *V* = 1 V or 1.25 V, a current was only detected in the target path but not in the sneak path, suggesting that at least one selector in the sneak path was not switched on. When *V* = 1.5 V, 1.75 V, or 2 V, the current through the target device was detected quickly after the rising edge of the voltage pulse. The sneak path current was detected much later. As all selectors have similar resistance in HRS, each of them should share around 1/3 of *V*, which was ≈0.5−0.7 V, corresponding to a delay of a few hundreds of microseconds to a few milliseconds, as shown in Figure 3b. After one of the selectors in the sneak path switches, the rest would switch faster than the first one due to increased voltage drop.

In addition to the floating scheme, the setup also works by grounding unselected BLs and WLs. Such a setup could be simplified because only devices that shared the same BL or WL with the target device could form the sneak paths. The other devices could not because both their top electrodes and bottom electrodes were grounded. The simplified circuit is shown in Figure 3c, where the positive and negative voltages were applied to BL1 and WL1, respectively, and WL2 was grounded. The currents through the target and sneak paths were monitored by 68 kΩ standard resistors. The voltage drop on a sneak path device was *V*/2. Target path and sneak path currents when *V* ranged from 1 to 2 V are shown in Figure 3d. It was observed in general that the sneak path current was detected sooner than the first setup because of a higher voltage drop on the sneak path selectors in this scheme. However, there was still a sufficient large gap between delays of the target device and the sneak path device, thanks to the exponential delay–voltage relation in Figure 2d. In a large array where all unselected WLs and BLs are grounded, due to the extremely high resistance of a timing selector in its OFF state, the combined sneak path currents through all the sneak paths would be negligible unless the number of devices connected to the same BL or WL of the target device is comparable with the ON/OFF ratio of the timing selector, which was more than 10^7^. In other words, this timing selector can support extremely large arrays. Because only devices sharing the same WL or BL as the target device are under the programming voltage, the scenario can be simplified as the case shown in Figure 3c.

**Figure 4:**
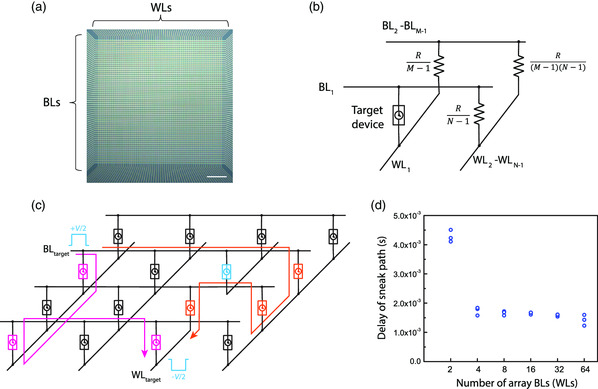
The sneak path delay in large timing selector arrays. a) The optical image of a 64 × 64 timing selector crossbar array. Scalar bar: 100 μm. b) The equivalent circuit of an M × N timing selector crossbar array, assuming that all the timing selectors are in their HRS and have the same resistance. c) Measurement schematic of the whole‐array sneak path delay in a customized large array. All the unlabeled wires are floating. The layout is designed so that part of the WL_target_ near the target device breaks. By applying positive/negative voltages to BL_target_/WL_target_, the device in blue, which should be the target device if the array is intact, cannot be switched because its bottom electrode is floating. However, multiple sneak paths can still be switched on if the applied pulse has sufficient amplitude and width. Two possible sneak paths are marked by pink and orange lines. The whole‐array delay is determined by the sneak path that can be switched on in the shortest time under a given voltage. d) The measured whole‐array delay time in different sizes of square arrays (the number of BLs is equal to that of the WLs) using the method in (c) when *V* = 1.2 V. Each circle represents one measurement event. The small fluctuation is due to the cycle‐to‐cycle variation of a timing selector.

To understand the sneak path behavior in a crossbar circuit where all unselected WLs and BLs are floating (or equivalently connected to high‐impedance terminals), a larger crossbar array with timing selectors was developed, as shown in **Figure** **4**a.

Assuming the wire resistance is negligible, and all the sneak path selectors in an *M* by *N* array have the same HRS resistance *R*, the equivalent circuit of sneak path devices can be simplified, as shown in Figure 4b, where all the sneak path devices connected to the same BL (WL) as the target device are equivalent to a resistor whose resistance was $\frac{R}{N-1}$ ($\frac{R}{M-1}$) and the other devices are equivalent to a resistor whose resistance was $\frac{R}{\left(N-1\right)\left(M-1\right)}$.^[^ ^20^ ^]^ In a very large array, $\frac{R}{\left(N-1\right)\left(M-1\right)}$ is much smaller than either $\frac{R}{N-1}$ or $\frac{R}{M-1}$; thus, these devices only share a small portion of the voltage *V*, which was the voltage drop on the target device. If *N* = *M* and *N* is much smaller than (*N* − 1)^2^, the devices on the same BL or WL as the target device share about *V*/2, which means that the sneak path delay will also be similar to the case shown in Figure 3c,d. If *N* >> *M*, $\frac{R}{M-1}$ is much larger than $\frac{R}{N-1}$ and $\frac{R}{\left(N-1\right)\left(M-1\right)}$. Due to the voltage‐dividing effect, voltage drops on sneak path devices on the same WL as the target device are close to *V*, which suggests that it is desirable to have *N* and *M* with similar values when designing timing selector crossbars.

To verify the earlier analysis, delays of sneak paths in timing selector crossbar arrays of different sizes and a square shape (the number of WLs equals that of BLs) were measured. Because it is impractical to measure all possible sneak paths simultaneously, the normal crossbar was modified in a way that the voltage input could only trigger sneak path currents with respect to a predefined target device but not activating the target selector itself. The crossbar design is shown in Figure 4c, where a segment of the WL electrode of the target device was removed, and the delay of the output current was determined by the sneak path with the shortest delay. The measurement results indicated that the sneak path delays in large arrays were all similar to that of a 4 × 4 array. In a 4 × 4 array, the estimated voltage drops on the sneak path devices sharing the same BL or WL with the target device were 3*V*/7, which was close to *V*/2, as in the case where all unselected BLs and WLs were grounded. When the array got larger, this voltage was even closer to *V*/2  but always a bit smaller. As a result, in the case where the unselected WLs and BLs are floating, and the numbers of WLs and BLs are the same, the delay difference between the sneak path and target device in a large time‐selector crossbar array is almost the same as the case where unselected WLs and BLs are grounded. In contrast, the larger the difference between the numbers of WLs and BLs, the less likely the timing selector can work with the floating scheme.

**Figure 5:**
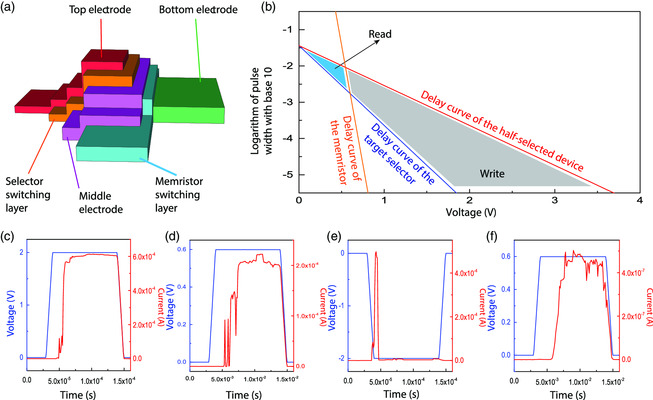
The electrical operation of a 1S1M integrated device. a) The schematic of a 1S1M device. The memristor uses Pt as the bottom electrode and Ta as the top electrode, whereas the selector uses Pt for both electrodes. The middle electrode consists of two layers: the Ta at the bottom that serves as the memristor top electrode and Pt at the top that serves as the selector bottom electrode. The resistive switching layer is HfO_2_ for the memristor and Ag‐doped SiO_2_ for the selector. b) The delay curve defined the operation condition of a 1S1M device. The delay curve of a selector (in dark blue) and a memristor (in orange) is shown according to the linear fitting of the logarithm of measured delay time versus voltage. The half‐slope delay curve (red) is plotted by changing the slope of the fit delay curve of a selector to half of it. If the amplitude and width of the input pulse lie in the region between the selector delay curve and the half‐slope delay curve, the target selector can be switched on, while the sneak path selectors cannot. If the amplitude and width of the input pulse are on the right side of the memristor delay curve, the memristor can be written into a new resistance state; otherwise, it can only be read by the pulse. c–f) Four basic operations of a 1S1M device: c) set; d) low‐resistance reading; e) reset; f) high‐resistance reading. Note that in all the operations the selector was always switched on from its off‐state first and then the applied voltage shifts from selector to memristor for corresponding operations. The difference in current scales in panels (d) and (f) is about three orders of magnitude, suggesting that the memristor has been successfully switched and read.

To verify if the sneak path‐blocking voltage pulse can also perform basic operations (set, reset, and read) to the 1S1M devices and the fabrication process compatibility between memristors and timing selectors, transient dynamics of both timing selectors and memristors were analyzed together and an integrated 1S1M device was fabricated for experimental demonstration, as shown in **Figure** **5**a. Memristor material stack and electrical behavior are shown in Figure S1, Supporting Information, and a timing selector was fabricated on top of the memristor. Here, the top electrodes of memristors consist of a Ta layer and a Pt layer, which could effectively prevent Ag from diffusing into the memristors.

The delay–voltage curves of a memristor and a timing selector were log fitted to experimentally measured data, as shown in Figure 5b. The fit delay–voltage relationship of the selector is log_10_ *t* = −2.2 *V* − 1.44, where *t* is time and *V* is voltage. Assuming that the voltage amplitude of a sneak path is half of that of the target device and their pulse width is the same, it could be inferred that a voltage signal with parameters (pulse amplitude, pulse width) that can successfully operate the target selector device without disturbing other devices in an array should fall between the selector's delay curve and another curve with the same intercept (log_10_ *t* = −1.1 *V* − 1.44) but a half slope, called delay curve of the half‐selected (half‐biased) selector, as shown in Figure 5b. Any of such voltage signals between these two curves would switch the target device because they are above the delay curve of the target selector, whereas they would not switch the half‐selected or partially selected selectors, because the parameters (pulse amplitude/2, pulse width) are below the delay curve of the half‐selected devices. Meanwhile, the delay curve of the memristor intersected with both curves. (Note that only the delay curve for set operation is shown for the sake of simplicity, whereas that for reset is similar but with opposite voltage polarity.) Like the selector, only a voltage input with parameters above the curve would switch the memristor. Thus, the parameters of a reading pulse and a writing pulse were limited in certain ranges, as shown in Figure 5b. It should be noted that during operations the voltage input will drop on the selector first and then shift to the memristor after the selector is switched on. The actual writing time is the summation of the delays of both the selector and the memristor, which means that the lower boundary of the writing region should be defined by the delay curve of a combined 1S1M unit. However, at a relatively high voltage (e.g., larger than 1 V), the memristor delay is negligible compared with that of a timing selector. As a result, the 1S1M delay curve should almost overlap with the selector‐only delay curve when the voltage is larger than 1 V.

Based on the result shown in Figure 5b, voltage pulses with customized parameters were applied to the integrated 1S1M cell and realized all basic functions, namely, set, reset, HRS reading, and LRS reading, as shown in Figure 5c‐f. As the chosen parameter was in the region below the half‐slope delay curve, no sneak path selector was expected to be switched. An important reason that memristor crossbar array can accelerate the calculation of the weighted sum of artificial synapses is that the calculation can be carried out with very high parallelism. Parallel operations of multiple memristors in a small 1S1M array with discrete timing selectors and memristors were experimentally verified (see Figure S2, Supporting Information, for more details).

## 3 Conclusion

The sneak path problem is a fundamental issue in crossbar arrays and can potentially be solved by introducing selectors into the arrays. In this work, the transition dynamics based on nonlinear delay–voltage relation of a selector and a memristor were proposed and demonstrated to tackle this problem. It was observed that the delay time of both selector and memristor is dependent on the voltage amplitude of the pulse input. As the voltage drop on the target device is larger than those on the sneak path devices, we conceptualize the timing selector with which we take advantage of a much shorter delay time of the target device than those of the sneak path devices to block the sneak path currents while passing the current through the target device. To achieve this target, a pulse with a specific amplitude was used, which could switch on the timing selector of the target cell but not those in the sneak paths within the pulse length. Integration of a timing selector and a memristor was realized, and its operating map was identified based on the experimentally measured delay–voltage relationships of both devices. While Ag‐based diffusive memristors were used as an example of the timing selectors for the proof‐of‐principle demonstrations, they are not necessarily the ideal devices for a timing selector. New types of timing selectors to be developed based on fast diffusive species with even lower activation energies, such as hydrogen and Li, etc., may lead to faster dynamics and more uniform switching that can further advance this new approach.^[^ ^34^ ^]^ Other material systems that can offer better uniformity from device to device and from cycle to cycle, as well as faster speed, are also interesting candidates to explore in the future.

## 4 Experimental Section

4.1

4.1.1

##### Device Fabrication

The bottom electrode of the selector was patterned by lithography using a negative photoresist on a SiO_2_ substrate. The photoresist on the electrode regions was resolved during development. 20 nm Pt was evaporated onto the whole substrate, followed by a lift‐off process that removed all the Pt on the photoresist and only kept the electrode region. The device was then cleaned by acetone/isopropyl alcohol that removed all the residual photoresist. Ag and SiO_2_ were then deposited onto the device by cosputtering at 100 W for 20 s and 270 W for 360 s, respectively. After SiO_2_ deposition, the top electrode was patterned and deposited, including the sputter of Ag at 100 W for 20 s and Pt at 200 W for 240 s. The deposition was followed by a lift‐off process and cleaning similar to the bottom electrode process. In the memristor part, the bottom electrode was evaporated Pt with a thickness of 20 nm. The resistive switching layer was a 5.5 nm Ta_2_O_5_ layer deposited by sputter. The top electrode was 50 nm Ta by sputter covered by a 10 nm Pd protection layer, also by sputter.

## Conflict of Interest

The authors declare no conflict of interest.

## Data Availability Statement

Research data are not shared.

## Supporting information

Supplementary Material
