# Supplementary material for: Timing Selector: Using Transient Switching Dynamics to Solve the Sneak Path Issue of Crossbar Arrays
Source: Small Sci. 2021 Oct 10;2(1):2100072. doi: 10.1002/smsc.202100072 (PMC11936054; doi:10.1002/smsc.202100072)
Supplement: Supplementary file 1 — Supplementary Material [file SMSC-2-2100072-s001.pdf]

# Timing Selector: using transient switching dynamics to solve the sneak path issue of crossbar arrays

*Mingyi Rao[1], Wenhao Song[2], Fatemeh Kiani[1], Shiva Asapu[1], Ye Zhuo[2], Rivu Midya[1], Navnidhi Upadhyay[1], Qing Wu[3], Mark Barnell[3], Peng Lin[1], Can Li[1], Zhongrui Wang[*,1], Qiangfei Xia, J. Joshua Yang[*,1,2]*

[1] Department of Electrical and Computer Engineering, University of Massachusetts, Amherst, Amherst, MA 01003, USA
[2] Ming Hsieh Department of Electrical and Computer Engineering, University of Southern California, 3740 McClintock Avenue, Los Angeles, CA 90089, USA
[3] Air Force Research Lab, Information Directorate, Rome, New York 13441, USA
*emails: jjoshuay@usc.edu, zrwang@eee.hku.hk.

## Supporting information

In the monolithically integrated 1S1M stack, the memristor material stack is schematically shown in Figure S1a, and its electrical behavior is shown in Fig. S1b.

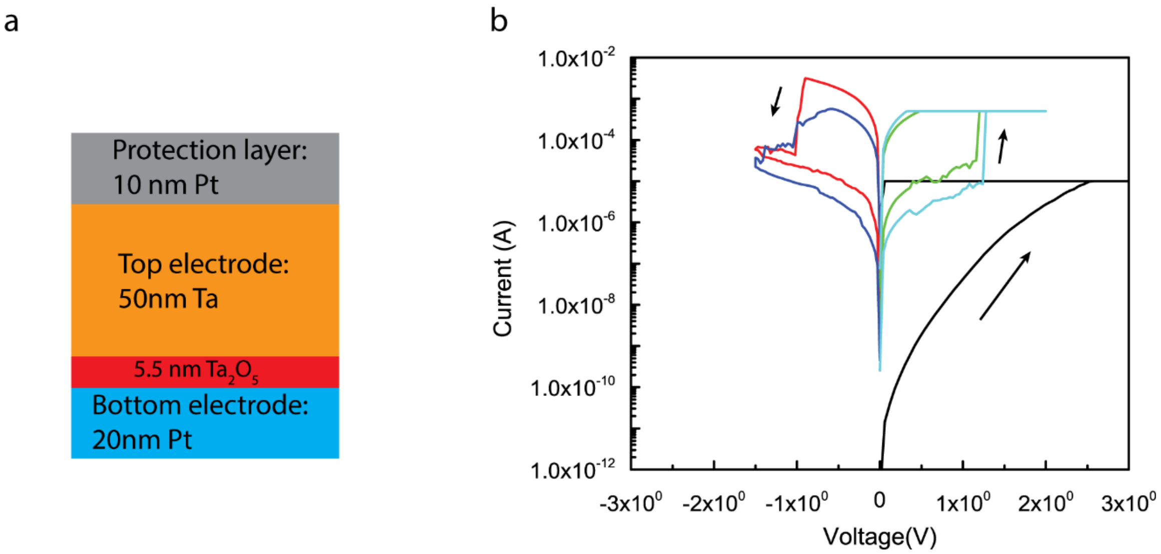


Figure S1 The memristor a) material stack and b) electrical characteristic. The black curve marked the forming process, and the others show repeatable set/reset cycles of the memristor in a).

To verify that integrating timing selectors into memristor crossbar array does not compromise the parallel reading capability, a 2 by 2 array was built with individual memristors and timing selectors, which were connected by cables and standard memristors to monitor current, as shown in Figure S2. To read the devices in WL1, negative voltage -$V$/2 was applied to WL1, and positive voltage $V$/2 was applied to BLs that were connected to target devices. The pulses were applied simultaneously, with a width of 1 ms. The untargeted WL (WL2) was grounded. When $V$ = 0.7V, only selectors in target paths were switched on while the selectors in sneak paths stayed in HRS, as shown in Fig. 6b, the results indicated that the state of memristors in the same WL (BL) could be measured simultaneously if each BL (WL) is connected with a current reading circuit. The summation of current from all BLs (WLs) to a target WL (BL) could be measured if there is a reading circuit in the WL (BL).

In a larger array, the two terminals of all the devices are connected to certain pairs of BL and WL. By applying appropriate voltage pulses to the chosen BLs and WLs and grounding the rest, only the target devices can be accessed, similar to the scenario in Figure S2a. Thus, the sneak path problem in a pure memristor array could be solved. It was also inferred that the timing selector was compatible with the vector multiplication operation. Fast vector multiplication by a memristor crossbar makes it a promising neuromorphic computing engine. According to Figure S2, all selectors in WL1 were switched on simultaneously so that the output at WL1 was the multiplication result of vector ($V_{\mathrm{BL1}}$, $V_{\mathrm{BL2}}$) and ($g_{\mathrm{PATH1}}$, $g_{\mathrm{PATH2}}$) where $g$ was the total conductance in its conducting path, which resembled the process of calculating the weighted sum of pre-synaptic signals in an artificial neural network.

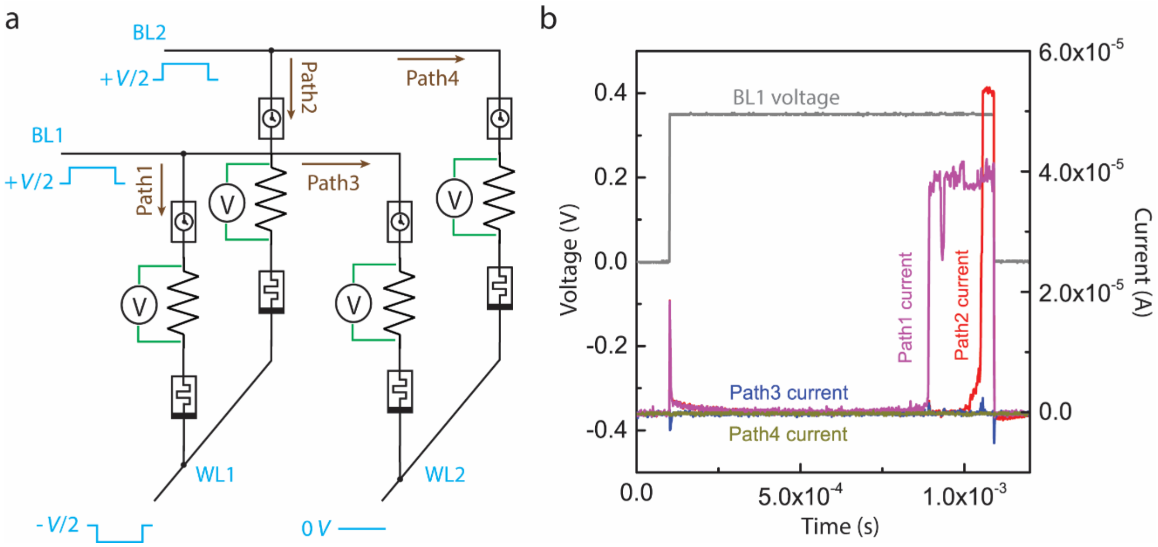


**Figure S2** Parallel reading of multiple memristors. (a) Individual Selectors, memristors, and 10 kΩ standards resistors are connected into a 2 by 2 array. Voltage pulses with the same width are applied to both BLs and WL1 simultaneously. The current in each path can be monitored by measuring the voltage drops on each standard resistor. (b) The measured current in all four paths in (a). BL1 voltage is also plotted as a reference, which is the same as BL2 input and in the opposite polarity of WL1 voltage. The selectors in path1 and path2 are switched on, making the memristor in these paths can be accessed. The small difference between the delays of path1 and path2 are caused by device-to-device variation in voltage-delay relationships. Since the difference between the delay of a sneak path and the target path can be orders of magnitude, such level of variation will not cause a failure in timing selectors functioning if the input parameters are carefully chosen according to Fig. 5b.

For the case of vector-matrix multiplication, where the inputs are typically represented by voltage pulses with different amplitudes, the voltage range needs to be linearly transformed according to the following plot so that all selectors of the underlying matrix are switched ON prior to sampling output currents. For example, here we employ pulses of width $T$, BL voltage $V_{BL}$, WL voltage $V_{WL}$. The selector/memristor delay curves are as shown below. Assuming pulse width $T$ corresponds to a minimum valid 1S1M reading voltage $V_1$ (limited by the timing selector delay curve, as shown in the figure below) and a maximum reading voltage $V_2$ (limited by memristor delay curve), the requirements can be described as follows:

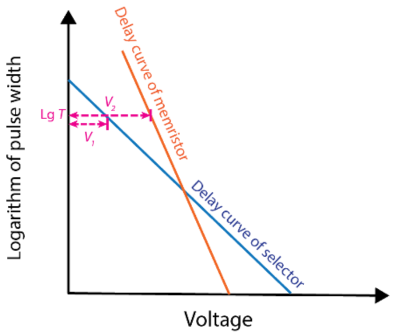


1. Duration $T$ of all input pulses that represent the vector should be same so that a vector matrix multiplication can be performed by reading the sum of output current at the end of the input pulses.
2. For every BL – WL pair that define a 1S1M cell, it is required that $V_1 < V_{BL} + V_{WL} < V_2$. This guarantees that the selector of the associated cell will be switched ON but the memristor of the associated cell will not be switched.
3. It is also required that both $V_{BL}$ and $V_{WL}$ are smaller than $V_1$. This guarantees that in the case of all unselected BLs and WLs are grounded, none of the unselected timing selectors can be switched to low resistance state.

The above scheme enables vector matrix multiplications on 1S1M arrays with analog input voltages.
